# Bringing human early embryo development to clinical interpretation: HESTA as an evolving reference

**DOI:** 10.1002/ctm2.70821

**Published:** 2026-09-03

**Authors:** Yuejiao Li, Ying Zhang, Haoqi Yan, Yiping Zhu, Minghui Yu, Xun Xu, Ya Gao

**Affiliations:** ^1^ State Key Laboratory of Genome and Multi‐omics Technologies BGI Research Shenzhen China; ^2^ BGI Research Shenzhen China; ^3^ BGI Research Wuhan China; ^4^ School of Life Sciences University of Chinese Academy of Sciences Beijing China; ^5^ College of Life Sciences Northeast Forestry University Harbin China; ^6^ Guangdong Provincial Key Laboratory of Genome Read and Write BGI Research Shenzhen China; ^7^ Shenzhen Engineering Laboratory for Birth Defects Screening BGI Research Shenzhen China; ^8^ Shanxi Medical University‐BGI Collaborative Center for Future Medicine Shanxi Medical University Taiyuan China

1

The regulatory mechanisms and cell‐fate decisions that govern early human development remain largely enigmatic. Many conditions diagnosed at birth or during childhood, including congenital malformations, neurodevelopmental disorders, and certain paediatric cancers, are thought to originate from transient molecular events occurring during organogenesis. To illuminate the fundamental principles of human embryogenesis and organ formation, we established the Human Embryo Spatiotemporal Transcriptomic Atlas (HESTA, https://db.cngb.org/hesta/), leveraging single‐nucleus RNA (snRNA) sequencing and spatial transcriptomic profiling of 77 sagittal sections derived from 13 euploid embryos spanning Carnegie stages (CS) 12–23.^1^ With unprecedented resolution, HESTA delineates 50 organs, 198 substructures, 607,093 nuclei, and 68 distinct cell types. This resource enables the reconstruction of canonical organ lineages and their regulatory programmes, as well as the mapping of disease‐associated gene expression patterns (Figure 1).

**FIGURE 1:**
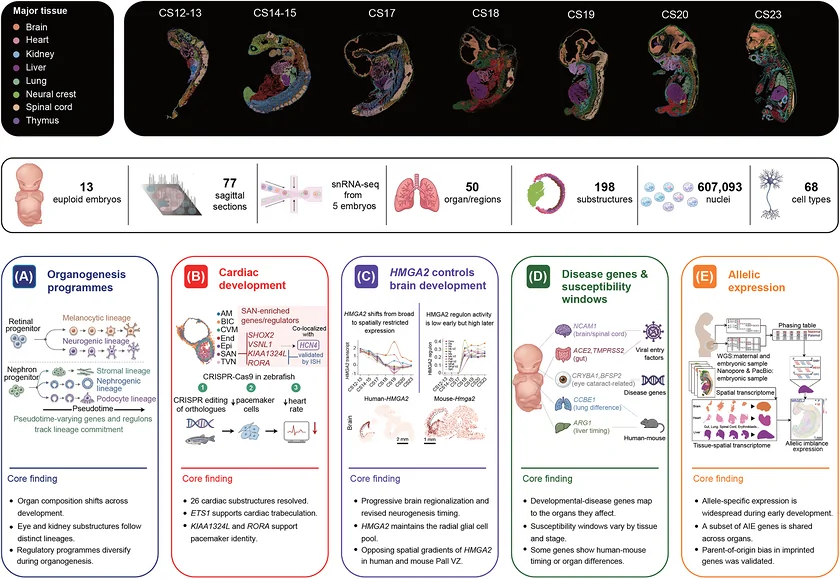
HESTA charts early human organogenesis and reveals disease‐relevant developmental programmes. The upper panel summarises the CS12‐23 whole‐embryo spatial atlas. Panels A–E highlight organ lineages and gene‐regulatory networks; cardiac substructures and pacemaker programmes; *HMGA2* regulation in brain lineage commitment; developmental‐disorder genes and infection‐related entry factors; and spatial allelic imbalance.

As a normative trajectory of human embryonic development, the atlas offers substantial value in identifying novel regulators of organogenesis, as shown in our original work by *KIAA1324L* and *RORA* in heart development, and *HMGA2* in brain development.^1^ The breadth and depth of the dataset further facilitate detailed investigations into less‐characterised tissues, including the eye, ear, nose, and oral structures. Through cross‐tissue comparisons of key transcriptional regulators, HESTA allows the discovery of both shared and tissue‐specific regulatory molecules. Another pivotal application lies in its utility as a normal transcriptome reference for diseased tissues, samples with developmental anomalies, and experimental models. Transcriptomic profiles derived from childhood tumours, congenital defects, or developmental abnormalities can be benchmarked against HESTA to uncover dysregulated genes, signalling pathways, and molecular markers linked to disease pathology (Figure 2).^2^ These comparisons can be further cross‐referenced with our original expression dynamics data for developmental disorder‐associated genes.^1^ Similarly, aligning single‐cell or spatial transcriptomic data from animal models or organoids with HESTA provides a quantitative measure of how faithfully these systems recapitulate human development. Such alignments play a key role in pinpointing the cell states, tissues, and developmental windows most suitable for follow‐up studies in organoids, animal models, or clinical cohorts, especially when investigating viral entry factors, drug targets, metabolising enzymes, transporters, and stress‐response pathways. This information is critical for generating testable hypotheses regarding disease susceptibility, maternal medication and therapeutic intervention (Figure 2).

**FIGURE 2:**
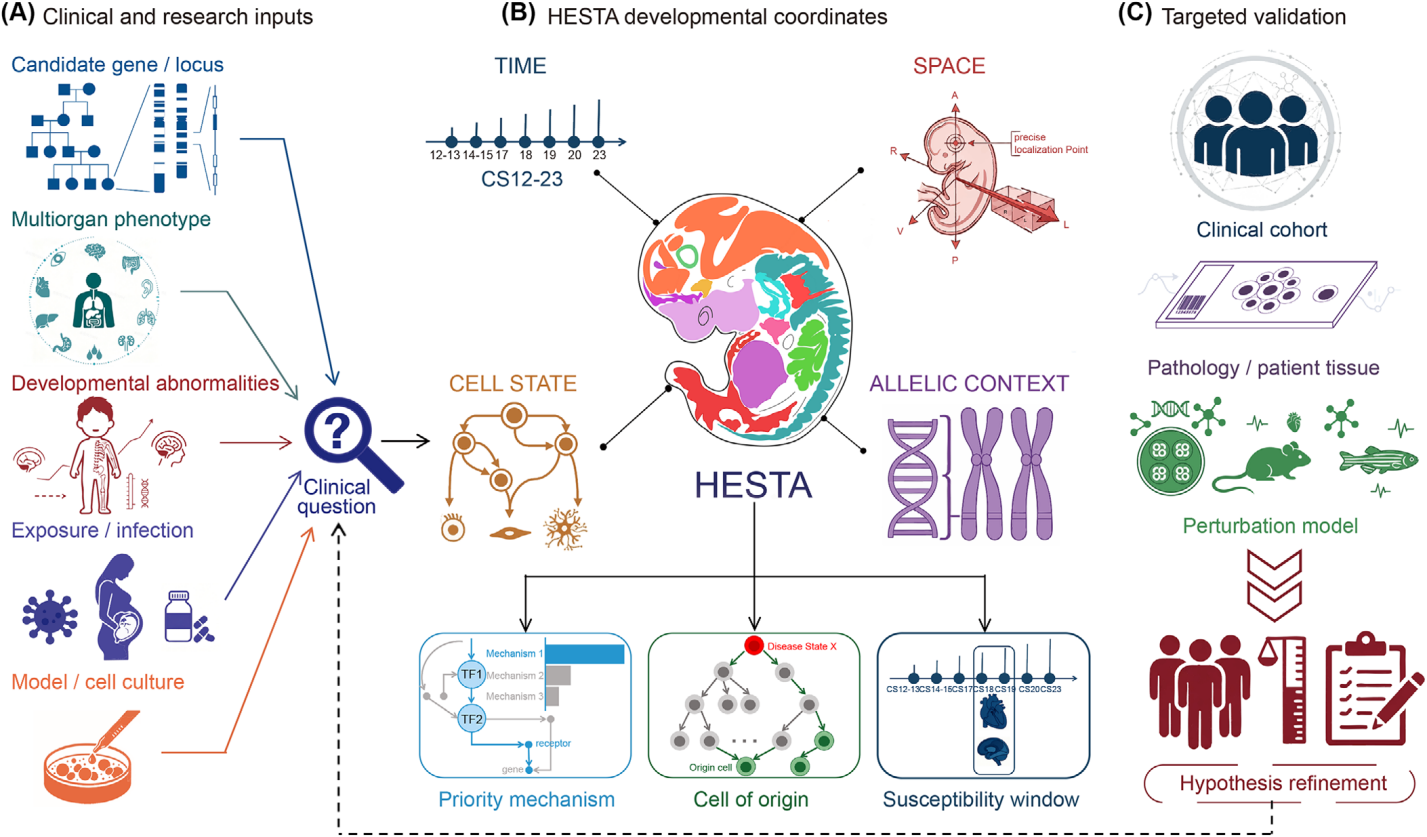
HESTA links clinical questions to developmental mechanisms. Clinical and research questions arise from candidate genes or loci identified in individual cases or population studies, incompletely characterised multiorgan phenotypes, developmental abnormalities, prenatal exposures or infections, and the need to assess how faithfully experimental models or cell cultures recapitulate normal human development. To address them, HESTA maps each question across developmental time, anatomical space, cell state and allelic context. This framework helps to prioritise candidate mechanisms, identify potential cells of origin and define windows of susceptibility. The resulting hypotheses are then tested in clinical cohorts, patient tissues or other pathological specimens and perturbation models, with the evidence generated used to refine the hypotheses and inform the original clinical question.

Moreover, HESTA serves as a powerful tool for identifying the cell types and organs in which disease‐associated genes and variants exert their effects. Traditional diagnostic approaches often detect pathogenic variants in relevant genes without explaining why a mutation manifests specifically in a particular cell type or tissue at a given developmental stage^3^. Through HESTA's online explorer, it is now feasible to map genetic findings from rare diseases, pregnancy loss, fetal or perinatal death, severe malformations, and even certain late‐onset disorders to their affected cell types and tissues, thereby prioritising the most plausible substructures, lineages, and developmental windows (Figure 2). In our original paper, we spatially and temporally anchored 1922 DDG2P disease genes, revealing not only organ‐specific patterns (e.g., *CRYBA1*/*BFSP2* in the eye) but also dual‐organ patterns, such as *SLC33A1*, *GFER*, and *FTL *in the eye and liver, as well as *TTN*, *LDB3*, and *LMNA *in the heart and skeletal muscle.^1^ Thus, projecting disease‐associated genes across organs may uncover multi‐organ phenotypes that are easily overlooked in routine diagnostic workups, enabling more targeted phenotypic assessments. Furthermore, population‐level genetic data such as genome‐wide association study (GWAS) outcomes can be overlaid onto single‐cell or spatial transcriptomic data to assess whether associated signals converge on shared developmental stages, substructures, lineages, or regulatory programmes. For instance, Alegbe et al. mapped GWAS risk variants for inflammatory bowel disease (IBD) to cell‐type resolution, revealing how non‐coding variants influence disease risk through specific cell populations.^4^ More recently, Song et al. developed gsMAP, a method that maps human complex‐trait GWAS signals onto spatial transcriptomic data, addressing the core question of which spatially localised cells mediate genetic risk^5^. Leveraging these novel approaches, HESTA will provide an essential reference framework for spatially localising genetic variants and identifying the cell types affected by disease‐associated mutations.

HESTA remains a dynamic and evolving resource. Expanding its coverage to include earlier and later developmental stages, reconstructing serial sections in three dimensions, and integrating chromatin accessibility, proteomic, metabolomic, lineage, genomic, and clinical phenotype data will enable the atlas to mature into a more comprehensive, interoperable reference for human development.^6^, ^7^ Such an endeavour will also require broader representation of diverse populations and disease‐relevant samples. As HESTA progresses, artificial intelligence (AI) and other emerging technologies could extend its utility beyond direct data exploration. For instance, models trained on paired haematoxylin and eosin (H&E) images and spatial‐omics data—such as GHIST—could infer selected spatial gene‐expression programmes, cell states, and regions of interest.^8^ Beyond image‐based inference, HESTA could provide a developmental context for gene‐regulatory network (GRN)‐driven or data‐driven perturbation models, aiding in the prioritisation of candidate genetic, transcriptional, or environmental perturbations for subsequent validation using Perturb‐seq, CRISPR‐based assays, organoids, or animal models (Figure 2).^9^ Ultimately, the same developmental framework could be incorporated into a HESTA‐constrained virtual‐cell model that represents a defined embryonic cell state by its developmental stage, anatomical location, lineage, regulatory network, and spatial context, and predicts its response to specified perturbations.^10^ Collectively, these strategies promise to establish HESTA as a common reference for both clinical and fundamental research questions spanning developmental biology and translational medicine.

## AUTHOR CONTRIBUTIONS

Yuejiao Li and Ying Zhang contributed equally to this work. Ya Gao, Yuejiao Li and Ying Zhang conceived the article.Yuejiao Li and Ying Zhang drafted the manuscript. Haoqi Yan, Yiping Zhu, and Minghui Yu made substantial contributions to the manuscript. Xun Xu and Ya Gao supervised the work. Ya Gao critically revised the manuscript. All authors reviewed and approved the final manuscript.

## CONFLICT OF INTEREST STATEMENT

Xun Xu is the key inventor of DNBSEQ and Stereo‐seq technologies covered in patents and patents applications, Xun Xu is the employee of BGI Genomics and holds stocks in BGI. Yuejiao Li, Ying Zhang, Haoqi Yan, Yiping Zhu, Minghui Yu, and Ya Gao are employees of BGI Research and hold no intellectual property and commercial interests in DNBSEQ and Stereo‐seq.

## DECLARATION OF GENERATIVE AI AND AI‐ASSISTED TECHNOLOGIES

During the preparation of this manuscript, the authors used ChatGPT (OpenAI, GPT‐5.6) and Gemini (Google, Gemini‐3.1) to assist with language editing and improve readability. These tools were also used to generate selected illustrative elements incorporated into the schematic figures. The AI‐generated visual elements were used solely for conceptual illustration and do not represent experimental data. All AI‐assisted text and visual content were subsequently reviewed, edited, and verified by the authors for scientific accuracy. The authors take full responsibility for the final content of the publication.

## Data Availability

Data sharing not applicable to this article as no datasets were generated or analysed during the current study.
